# Defining the Characteristics of Story Production of Autistic Children: A Multilevel Analysis

**DOI:** 10.1007/s10803-023-06096-2

**Published:** 2023-09-01

**Authors:** Ines Adornetti, Alessandra Chiera, Daniela Altavilla, Valentina Deriu, Andrea Marini, Marika Gobbo, Giovanni Valeri, Rita Magni, Francesco Ferretti

**Affiliations:** 1 Cosmic Lab, Department of Philosophy, Communication and Performing Arts, Roma Tre University, Via Ostiense 234-236, 00146 Rome, Italy; 2 Department of Languages and Literatures, Communication, Education and Society, University of Udine, Via Margreth, 3, 33100 Udine, Italy; 3 Claudiana - Landesfachhochschule Für Gesundheitsberufe, Bozen, Italy; 4 Department of Life Sciences, University of Trieste, 34127 Trieste, Italy; 5 Child and Adolescent Neuropsychiatry Unit, Department of Neuroscience, The Bambino Gesù Children’s Hospital, IRCCS, Piazza di Sant’Onofrio 4, 00165 Rome, Italy; 6 Studio Polispecialistico Evò, Viale Pier Luigi Nervi 164, 04100 Latina, Italy

**Keywords:** Autism spectrum disorders, Microstructure, Macrostructure, Narrative, Storytelling, Theory of mind, Working memory

## Abstract

Several studies suggest that a valuable tool to examine linguistic skills in communication disorders is offered by procedures of narrative discourse assessment. Following this line of research, we present an exploratory study aimed to investigate storytelling abilities of autistic children to better define the characteristics of their story production. Participants included 41 autistic children and 41 children with typical development aged between 7.02 and 11.03 years matched on age, gender, level of formal education, intelligence quotient, working memory, attention skills, theory of mind, and phonological short-term memory. Narrative production was assessed by analysing the language samples obtained through the “Nest Story” description task. A multilevel analysis including micro- and macro-linguistic variables was adopted for narrative assessment. Group differences emerged on both micro- and macro-linguistic dimensions: autistic children produced narratives with more phonological errors and semantic paraphasias (microlinguistic variables) as well as more errors of global coherence and a fewer number of visible events and inferred events (macrolinguistic variables) than the control group.This study shows that even autistic children with adequate cognitive skills display several limitations in their narrative competence and that such weaknesses affect both micro- and macrolinguistic aspects of story production.

## Introduction

The latest edition of the Diagnostic and Statistical Manual of Mental Disorders (DSM-5, APA, 2013) defines Autism Spectrum Disorders (ASD) as a heterogeneous group of neurodevelopmental disorders that can be described, at a behavioural level, by impairments in two main domains: persistent deficits in social communication and interaction; restricted, repetitive patterns of behaviour, interests, or activities. Because of their heterogeneity, in the diagnosis of ASD individual characteristics are determined with reference to some specifiers, including the one relating to the presence or absence of language impairment (APA, 2013; Rosen et al., 2021). Linguistic skills are, indeed, extremely variable among persons diagnosed with ASD (Arciuli & Brock, 2014; Boucher, 2012; Harper-Hill et al., 2013).

To define the linguistic profile of autistic persons, different language levels (e.g., structural language, semantic, and pragmatics) should be considered. As for structural language processes (e.g., phonology and syntax), some people have age-appropriate language skills (Naigles et al., 2011; Tek et al., 2014), while others show noticeable difficulties (Eigsti et al., 2007; Tuchman et al., 1991; Wittke et al., 2017). For this reason, structural language impairment in autistic persons has sometimes been regarded as a separate comorbid dysfunction (Arciuli & Brock, 2014). When it comes to semantic and pragmatics levels, impairments appear more common than structural language deficits, even among individuals who are within the normative range regarding cognitive and structural language skills (Kamio et al., 2007; Tager-Flusberg et al., 2005). Kamio et al. (2007) analysed semantic priming effects in children and adolescents with high-functioning autism (participants had to decide whether letter strings were words or non-words trying to answer as quickly as possible), finding an atypical automatic semantic processing in highly verbal autistic individuals (see also, Coderre et al., 2017; Henderson et al., 2011; McCleery et al., 2010; Pijnacker et al., 2010; Toichi & Kamio, 2001). Pragmatic impairment is a widely recognized feature of ASD that is independent of language level, age, and Intelligence Quotient (IQ) (Cardillo et al., 2021; Geurts & Embrechts, 2008; La Valle et al., 2020; Loukusa & Moilanen, 2009; Noens & Berckelaer-Onnes, 2005). Since pragmatic processing affects a wide range of linguistic phenomena, the difficulties in using language in context observable in autistic people are manifold. They include interpretation of figurative language (Happé, 1993; Whyte et al., 2014), understanding presuppositions (Cheung et al., 2020), comprehending humour and indirect requests (Emerich et al., 2003; Ozonoff & Miller, 1996), and making inferences about implied meaning in context (Dennis et al., 2001; Dindar et al., 2022; Loukusa & Moilanen, 2009).

Several lines of research suggest that a valuable tool to examine how these different linguistic skills interact in the communication of autistic people—and more in general, in communicative disorders—is offered by procedures of narrative discourse assessment (e.g., Kenan et al., 2019; Marini et al., 2014, 2020; Peristeri et al., 2017; Stirling et al., 2014). At a general level, a narrative discourse can be defined as a set of temporally and causally connected sequences of events, determined by the goals and motives of one or more characters, which unfold toward a conclusion and can be conveyed through different expressive systems (e.g., verbal, visual, etc.) (Adornetti et al., 2022). When processing a narrative, a complex set of skills is in place. For example, the single events that form a story must be coherently integrated into a higher-order sequence by establishing spatial and temporal links between them (Adornetti et al., 2020; Ferretti et al., 2018). Moreover, the construction of a story requires the integration of the different perspectives and psychological states of the characters and possibly the narrator (Chiera et al., 2022; Stirling et al., 2014). Overall, as narrative processing is a complex cognitive task drawing on multiple levels of language comprehension and production, its assessment is pivotal when analysing language and communication in clinical populations.

In the light of these considerations, we present an exploratory study aimed to investigate the characteristics of narrative processing, specifically story production, in autistic children. From a linguistic perspective, analysing story production allows investigating how two levels of processing combine each other’s: micro- and macrolinguistic processes (Davis & Coelho, 2004; Marini & Carlomagno, 2004). Microlinguistic processes refer to the microstructure of a narrative, i.e., to the production of the story at the level of individual sentences (within-sentence processing). Macrolinguistic processes refer to the macrostructure of a narrative, i.e., to the overall organization of story content (between-sentence processing), ensuring the pragmatic functionality of narrative. In the next section, previous research that has examined these two levels of processing in the narrative production of autistic people will be reviewed.

### Storytelling Abilities of Autistic Children

Investigations that have explored the microstructure of a narrative in ASD have mainly focused on variables such as the number of words and utterances (i.e., length of narratives) and syntactic complexity (e.g., percentage of subordinate sentences), and obtained mixed results (for a review: Baixauli et al., 2016; see also Stirling et al., 2014). Some studies did not find differences between the stories produced by autistic children and those generated by children with typical development (TD) in terms of story length and syntactic complexity (e.g., Diehl et al., 2006; Losh & Capps, 2003; Norbury & Bishop, 2003). However, other investigations showed that children with a diagnosis of ASD may display delayed morphosyntactic development (Park et al., 2012) resulting in the generation of narratives that were shorter (e.g., Kenan et al., 2019; Rumpf et al., 2012) and syntactically less complex (Capps et al., 2000; Peristeri et al., 2017; Stirling et al., 2017) than those produced by children with TD.

When narratives are examined in terms of macrolinguistic processing (e.g., representation of the story gist, organization of coherent chains of events, and complexity of the story structure) differences between storytelling abilities of autistic children and those of children with TD are more noticeable (e.g., Ferretti et al., 2018; Losh & Capps, 2003; Marini et al., 2019; Norbury & Bishop, 2003; Peristeri et al., 2017). Some studies showed that autistic children may experience difficulties ordering narrative information at a global level and that such difficulties depend on problems in managing the causal relationships among the events of a story (King et al., 2013, 2014; Losh & Capps, 2003; Sah & Torng, 2015). Diehl et al. (2006) assessed story recall and narrative coherence in 17 high-functioning autistic children and 17 peers with TD who were asked to listen to and retell the story *Frog, Where Are You?* (Mayer, 1969). Although no differences between the two groups emerged in story length and syntactic complexity, the autistic group produced stories that were significantly less coherent, i.e., with fewer causal relationships between linguistic units, than the control group. Ferretti et al. (2018) examined the ability of 66 high-functioning autistic children to generate fictional stories and found that their narratives contained fewer causal links and more errors of global coherence (measured in terms of tangential and conceptually incongruent utterances) than the stories produced by a group of participants with TD.

Other investigations analysing macrostructure focused on story structure complexity, which was often measured in reference to the use of internal states terms (ISTs) (i.e., verbal reference to mental states and emotions) and the production of units of information involving the crucial story elements (e.g., settings, characters, objects, events) (Kenan et al., 2019; Peristeri et al., 2017; Rumpf et al., 2012; Suh et al., 2014). The former variable (ISTs) is usually included in procedures of narrative discourse assessment in ASD because it is considered a linguistic measure of Theory of Mind (ToM), the cognitive ability to recognize and attribute intentions, emotions, and thoughts to others (Frith & Frith, 2005). Indeed, it has been suggested that the common difficulties of autistic persons in inferring the mental states and emotions of others (Baron-Cohen, 1999; Fletcher-Watson & Happé, 2019; Frith, 2003; Gauthier et al., 2009) may be responsible for their problems in understanding the goals and motivations underlying the actions of the story characters (e.g., Happé, 1994; Jolliffe & Baron-Cohen, 1999). Studies focusing on the use of ISTs during story generation have produced mixed findings. Some investigations reported that autistic participants used fewer mental state terms than the control groups when asked to tell a story (Losh & Capps, 2003; Peristeri et al., 2017; Rumpf et al., 2012), whereas other research failed to find group differences on this variable (Capps et al., 2000; Norbury & Bishop, 2003). As for investigations focusing on the inclusion of units of information relating to the main story elements, Kenan et al. (2019) found that 24 male autistic children had difficulties in managing the semantic-pragmatic dimension of story structure as they tended to include a smaller number of settings, characters, and actions (as well as fewer ideas related to ToM) than children with TD.

Overall, the existing literature on narrative production in ASD is characterized by heterogeneous results that may be due to different reasons. First, it is reasonable to hypothesise that inconsistency in findings might be partly imputable to the different narrative tasks that have been used in the literature and that might elicit capacities that do not completely overlap. For example, some investigations elicited storytelling employing visual narratives, such as wordless picture stories or sequence of picture cards (e.g., Kauschke et al., 2016; Kenan et al., 2019; Losh & Capps, 2003; Marini et al., 2020; Peristeri et al., 2017; Rumpf et al., 2012); others opted for retelling tasks (e.g., Diehl et al., 2006; Kimhi et al., 2022); further studies used prompts, i.e., provided children with a few story events and then invited them to imagine and tell the others (e.g., Ferretti et al., 2018; King et al., 2014; Marini et al., 2019). A further element complicating the picture is that many of these previous studies had small sample sizes. In the literature review by Baixauli et al. (2016), the mean number of autistic participants of the studies included in the publication was 18.54 (details are reported in Baixauli et al., 2016, pp. 239–243). Moreover, differences in the choice of the control group, i.e., in how participants of the comparison groups were matched to the autistic groups, should be contemplated. In some studies, autistic groups and control groups were matched for expressive and/or receptive language (e.g., Diehl et al., 2006; King et al., 2014; Peristeri et al., 2017), while in others not (e.g., Losh & Capps, 2003; Norbury & Bishop, 2003; Rumpf et al., 2012).

In the current exploratory study, we analysed storytelling abilities in autistic children with the aim of providing further evidence about the characteristics of their story production. Considering the problems affecting previous research, as for story elicitation we selected one of the most common paradigms used in the literature, i.e., sequence of picture cards, with participants asked to narrate the story. Moreover, we recruited a larger cohort of autistic participants than past research. We also controlled for possible linguistic differences between autistic children and participants of the control group using a non-word repetition task, which has been considered to be extremely sensitive to detect the presence of such differences (Harper-Hill et al., 2013; Kjelgaard & Tager-Flusberg, 2001; Marini et al., 2020). For example, Marini et al. (2020), observing that autistic children showing difficulties on a non-word repetition task also obtained the lowest scores on linguistic and narrative measures, hypothesized that such a task could be efficient in detecting the presence of differences in linguistic measures between groups of participants with a diagnosis of ASD and typically developing participants.

To provide an assessment of story production as comprehensive as possible, we included in our investigation both micro- and macro-linguistic variables. As for macrolinguistic dimension, we construed a list of variables integrating different approaches to analysing narrative, which are rarely used in a single study. Specifically, we aimed to put together approaches assessing how story’s main events are conveyed with approaches evaluating causal/temporal connections as well as narrative coherence. Lastly, we aimed to explore the role of two cognitive abilities in narrative production of autistic children: ToM and working memory. Previous studies found that they are both associated with many narrative skills, suggesting that these cognitive mechanisms explain some of the impairments in narrative production of autistic persons (e.g., Gabig, 2008; Kuijper et al., 2017). As mentioned, ToM allows the understanding of the psychological/motivational causes of characters’ actions and therefore might have a role in processing character-related information during narrative production. Working memory has been connected to a crucial process of narrative, i.e., events representation (e.g., Radvansky & Copeland, 2001; Radvansky & Zacks, 2014): when people represent a single event, they must keep track of the various aspects involved in that event as well as to integrate those aspects with information coming from both the environment and their world knowledge. Previous studies in both children with TD and autistic children found that working memory development correlates with several aspects of story production, including narrative length (e.g., Kuijper et al., 2017; Tsimpli et al., 2014). Based on the existing literature, we predicted that autistic children would show difficulties on micro-linguistic measures as well as in managing the story macrostructure, i.e., including fewer references to the crucial narrative elements, and in building coherent links between story’s events.

## Materials and Methods

### Participants

Eighty-two Italian-speaking children aged between 7.02 and 11.03 years were included in this study. They formed a group of children with a diagnosis of ASD and one of children with TD. The two groups were matched on chronological age, gender, level of formal education, and IQ level, as assessed through the Raven’s Coloured Progressive Matrices, which was in the normative range (Raven, 1938; Italian standardization: Belacchi et al., 2008) (Table 1).

**Table 1:** Descriptive statistics of the two groups (ASD and TD) of participants

	ASD (*n* = 41) M (SD) [range]	TD (*n* = 41) M (SD) [range]
Age	9.34 (0.95) [7.07–11.03]	9.13 (1.00) [7–02−11.03]
Education	2nd–5th grade	1st–5th grade
Gender distribution	Males = 32 (78%) Females = 9 (22%)	Males = 32 (78%) Females = 9 (22%)
IQ level (Raven)	109.76 (10.84) [90–130]	111.22 (11.44) [90–130]
ADOS-2 severity index	5.83 (1.41) [3–8]	–

Data are expressed as means, standard deviations, and ranges. Legend: *ASD* Autism Spectrum Disorders group, *TD* Typical Development group, *IQ* Intelligent Quotient, *ADOS-2* Autism Diagnostic Observation Schedule 2nd edition

The cohort of autistic children consisted of 41 children (32 boys and 9 girls) recruited at the “Bambino Gesù” Children Hospital in Rome. The diagnosis of ASD was established by clinical observation in compliance with the DSM-5 criteria (APA, 2013) by neuropsychiatrists of the Child and Adolescent Neuropsychiatry Unit at the “Bambino Gesù” Children Hospital in Rome. To determine the severity of the autistic symptomatology, the *Autism Diagnostic Observation Schedule 2nd edition* (ADOS-2; Lord et al., 2013) was employed. The severity scores were based on the standardized Calibrated Severity Scores (CSS), module 3, which range from 1 to 10. Overall, the group of autistic children had a mean severity score of 5.83 with a standard deviation of 1.41 ranging from 3 to 8.

The control group was formed by 41 children (32 boys and 9 girls) with TD recruited in local schools. In a preliminary interview, their teachers confirmed that they had normal cognitive development, as well as average school performance. According to parents’ reports, none of them had a known history of psychiatric or neurological disorders, learning disabilities, hearing, or visual loss.

This study was approved by the ethical committee of the “Bambino Gesù” Children Hospital in Rome. Parents signed the consent form for the participation of their children to the study and for the treatment of the data. Children were told that if they got tired or bored when doing the tests, they could withdraw at any time. None of them asked to withdraw.

### Procedure

The autistic children were tested individually at the “Bambino Gesù” Children Hospital in Rome. The typically developing children were tested individually at school in a quiet room.

Since previous investigations (Harper-Hill et al., 2013; Kjelgaard & Tager-Flusberg, 2001; Marini et al., 2020) showed that tasks of non-word repetition are extremely sensitive to detect the presence of linguistic differences between autistic children and typically developing children, participants were administered a test of non-word repetition to control for such possible differences. Additional tasks assessed working memory and ToM. Moreover, to verify that the two groups did not differ in their attention skills, a test aiming at evaluating selective and sustained attention was administered. Finally, they were all asked to perform a story description task.

All tasks were administered on the same day. The battery was about one hour long. Children were told that they could have a break to rest, but none of them asked for it. The tasks were administered to all participants in the same order, which was the following: Raven’s Coloured Progressive Matrices, non-word repetition, working memory, attention skills, theory of mind, story description task. As for the autistic group, data about CSS from ADOS-2 were extracted from medical records of the “Bambino Gesù” Children Hospital in Rome.

### Assessment of Narrative Production

Narrative production was assessed by analysing language samples obtained during a narrative production task of the Batteria per la Valutazione del Linguaggio in bambini dai 4 ai 12 anni (BVL_4-12; Marini et al., 2015), a standardized battery of tests designed to assess language development in children aged from 4 to 12 years old. Namely, the participants were shown a cartoon story made of six drawings presented on the same page (the “Nest Story” originally by Paradis, 1987). The experimenter told them: “*Now you will see a picture story. I don’t know this story. You must tell me it. There is no right or wrong way to tell it. You can talk a lot or a little. I only ask you not to use words such as “here” or “this”. Try to be clear*.”

Administration and transcription procedures followed the criteria outlined in Marini et al., (2011a, 2011b). Each story was audio-recorded and subsequently transcribed verbatim by three trained students who were in their final stage of the master’s degree of Cognitive Sciences of Communication at Roma Tre University and who did not know which group participants belonged to. The transcriptions included phonological fillers, pauses, false starts, and extraneous utterances. These transcriptions were manually compared to obtain highly reliable texts for analysis. Discrepancies were discussed and resolved before the narratives were analysed further.

Narrative assessment was conducted adopting a multilevel procedure including micro- and macro-linguistic variables. As for microstructure, five measures often employed in the literature (Baixauli et al., 2016) were used: the *number of words* and the *number of utterances*, which were considered indicators of narrative length; the *percentage of subordinated clauses* included in the narratives, which was considered as a measure of syntactic complexity; the percentage of *phonological errors,* which was considered assessing the participants’ ability to retrieve phonologically well-formed words (Marini et al., 2011a, 2011b)*;* the percentage of *semantic paraphasias,* which was considered as an indication of lexical-semantic processing (Andreetta et al., 2012).

As for narrative length, we rated both the number of words and the number of utterances. As for the number of words, the total number of well-formed words was calculated for each story. To compute the number of utterances, each story was segmented into utterances, and the total number of utterances (including those containing unintelligible words) was assessed following criteria detailed in Marini et al., (2011a, 2011b) and Ferretti et al. (2018). Accordingly, we adopted several criteria for segmenting text into utterances: acoustic, semantic, grammatical, and phonological ones. As explained by Marini et al. (2011, p. 1379), since “it is hardly possible to provide a coherent segmentation by adopting just one criterion”, it is desirable to jointly adopt acoustic, semantic, grammatical, and phonological criteria. According to the acoustic criterion, an utterance is an emission of phonemes delimited by pauses that can be easily identified. Let’s consider the following sequence: “*ci sono … (silent pause of 3 s) una donna e un uomo* (“there are … a woman and a man”). In this case, since a clear pause can be perceived between the first chunk “*there are*” and the second one “*a woman and a man*,” the sequence can be segmented in two distinct utterances: /there are/a woman and a man/. According to the semantic criterion, an utterance is a conceptually homogeneous piece of information—i.e., a proposition, defined as a semantic unit consisting of the main predicate with its arguments and all embedded predicates and argument(s) associated with it. For example, the sequence “*Ci stanno un signore e una signora che stanno fissando un nido con un uccello. Poi il signore si arrampica”* (“There are a man and a lady who are staring at a nest with a bird. Then the man climbs up”) can be split in two distinct utterances: /There are a man and a lady who are staring at a nest with a bird/Then the man climbs up/. According to the grammatical criterion, a set of words can be considered an utterance when, in absence of clear pauses (acoustic criterion) and of propositional violations (semantic criterion), it forms a grammatically complete sentence (eventually also including subordinate clauses). For example, the sequence “*il ragazzo decide di arrampicarsi sull’albero per prendere il nido di uccelli* (“the boy decides to climb the tree to get the bird's nest”) can be considered a single utterance. However, if the speaker utters two or more coordinated sentences, such as “*il ragazzo decide di arrampicarsi sull’ albero per prendere il nido di uccelli ma il ramo si spezza e cade”* (“the boy decides to climb the tree to get the bird's nest but the branch breaks and he falls”), these can be divided in three separate utterances: /the boy decides to climb the tree to get the bird's nest/but the branch breaks/and he falls/. Lastly, the phonological criterion allows dividing the utterances when there is a phonological interruption between them: an utterance is considered abruptly interrupted when it contains an interrupted word (i.e., there is a false start). For example, the sequence “*una signora e un sig- …e un ragazzo”* (/ a lady and a gent- /… and a boy/) can be split in two distinct utterances. In the statistical analyses, the total number of utterances was considered.

To assess syntactic complexity, for each story the total number of subordinated clauses used by the participants in their narratives was calculated. A subordinated clause is a clause that cannot stand alone as a complete sentence but is linked to the main clause by a subordinating conjunction. For example, in the sequence *il ragazzo decide di arrampicarsi sull’albero per prendere il nido di uccelli* (“the boy decides to climb the tree to get the bird's nest”), the subordinated clause is *per prendere il nido di uccelli* (“to get the bird's nest”). Then, the percentage of subordinated clauses was calculated by dividing the total number of such clauses by the number of utterances and then multiplying by 100.

The percentage of phonological errors was calculated following the criteria described in Andreetta et al. (2012). False starts, phonological and phonetic paraphasias and neologisms were counted as phonological errors. To compute the percentage, the total number of phonological errors was divided by the number of units (each word, non-word or syllabic false start uttered by the speaker) and then multiplying this value by 100.

The last micro-linguistic variable was that measuring the percentage of semantic paraphasias, which represent a way to evaluate children’s ability to select semantically appropriate words. Following the criteria described in Andreetta et al. (2012), when a target word was replaced by a semantically related word a semantic paraphasia was counted. For example, in the following sequence *la mamma chiama l’ambulanza* (“the mother calls an ambulance”), the word *mamma*/mother was considered as a semantic paraphasia as the speaker implied *moglie/*wife. Lexical-semantic processing was measured in terms of the percentage of occurrences of semantic paraphasias on the total number of content words. Higher values represent more semantic errors per word.

As for macrostructure, to assess the children’s ability to construct a global representation of the narrative, the analysis focused on the units of information produced to convey the essential story components, i.e., the *core story details;* the cohesive connectives linking the story events according to causal and temporal principles, i.e., *first–second-third order connectives* and *temporal markers*; and the percentage of *local* and *global coherence errors*. Moreover, the analysis also included a variable evaluating children’s ability to infer implicit events, i.e., *inferred events* and a last variable evaluating the children’s ability to interpret the characters’ mental states and emotions, i.e., *internal states terms.*

As for the *core story details*, a list of measures was created adapting a semantic-pragmatic evaluation employed by Kenan et al. (2019). In particular, the list included the following categories:

*Settings* The number of references to settings where the story events take place was counted for each participant. A list of these items was prepared in advance; a total of 4 settings were established: garden/house/ambulance/hospital. Synonyms were scored as correct (e.g., ‘park’ instead of ‘garden’).

*Objects* The number of references to concrete objects that are visible from the pictures of the story was counted for each participant (e.g., nest, bed, pillow, window).

*Characters* The number of references to the story characters was counted for each participant. A list was prepared in advance: 9 individual characters were present in the story (3 birds and 6 persons).

*Visible events* This measure focused on the children’s use of clauses to refer to concrete events that were visible from the pictures of the story, requiring the direct interpretation of the visual stimuli. An example of visible event was the following: *Ci stanno un signore e una signora che stanno fissando un nido con un uccello* (“There are a man and a lady who are staring at a nest with a bird”). The number of visible events generated in the children’s narratives was counted for each participant.

To assess the children’s ability to connect the story events, the use of connectives (e.g., *because*, *and then*) serving to signal the causal and temporal relations between sentences was counted. In particular, we evaluated the generation of cohesive elements used to mark different levels of discourse: (a) *first-order connectives*: connective elements used by children to link events that were included in the same drawing of the six frames comprising the cartoon story; this use would reflect the processing of local stimulus properties; (b) *second-order connectives*: connectors used by children to connect events referring to two distinct drawings of the cartoon story; this use would require the ability to relate to information conveyed in previous pictures by interpreting a variety of story details; (c)* third-order connectives*: connectors used by children to link two events, of which at least one was not present in the stimuli, i.e., inferred event; this use would reflect a complex integration of story details into meaningful wholes to construct a coherent representation of the narrative scene. The number of connectives, for each type, used in the children’s narratives was counted for each participant.

As for the temporal connections between events*,* we assessed the usage of *temporal markers*: the number of indicators used to signal the temporal relationships between events, e.g., *soon*, *later*, was counted for each participant.

To determine the extent to which each utterance of the story was conceptually related to the previous one, we measured local coherence. Following the criteria described in Andreetta et al. (2012), we evaluated *local coherence errors*, which included the production of words without a clear referent and topic switching. The percentage of local coherence errors was calculated by dividing the number of local coherence errors by the number of utterances and multiplying this value by 100.

To determine the extent to which each utterance of the story was conceptually related to the main topic of the story, narrative global coherence was evaluated. Also in this case, we counted the percentage of *errors of global coherence* (Andreetta et al., 2012). Errors of global coherence included the production of utterances that may be tangential (containing a derailment in the flow of discourse with respect to the information provided in previous utterances), conceptually incongruent with the story (including ideas not directly addressed by the stimulus), propositional repetitions or simple fillers. The percentage of global coherence errors was calculated by dividing the number of global coherence errors by the number of utterances and multiplying this value by 100.

The children’s ability to infer implicit events, i.e., events that were not apparent in the stimuli, from the integration of story details in a relevant and accurate fashion was assessed measuring *inferred events*. For example, the fourth drawing of the story depicts a broken branch, the nest with the birds on the ground, a man lying on the ground with a broken leg, and in the background three people pointing at the man. In the fifth scene, the same man has a bandaged leg and is on the stretcher about to be loaded into an ambulance. The event that must be inferred to coherently connect these scenes is that someone called an ambulance. Therefore, an utterance such as *chiamarono l’ambulanza* (“They called an ambulance”) was considered as *inferred event.* The number of inferred events generated in the children’s narratives was counted for each participant.

The children’s ability to mention the characters’ emotional and cognitive states was measured in terms of *internal states terms* (ISTs): the number of unique lexical items expressing negative or positive emotions (e.g., *sad*) and mental state verbs (e.g., *think, wonder*) (Peristeri et al., 2017), was counted for each participant.

Both micro-and macrolinguistic analyses were performed independently by two trained students (who were different from those who transcribed the stories) attending the final year of the master’s degree of Cognitive Sciences of Communication at Roma Tre University who knew the main aim of the study but did not know which group the children belonged to. Micro-and macrolinguistic analyses resulted in substantial agreement: the inter-coder reliability for macrolinguistic variables was 0.69 < r < 0.91; p < 0.001; for macrolinguistic variables was 0.48 < r < 0.90; p < 0.001 (only first-order connectives resulted low agreement *r* = 0.28; p = 0.011). Discrepancies were resolved through discussion by the two evaluators.

### Assessment of Phonological Short-Term Memory

The non-word repetition subtest of the *Prove di Memoria e Apprendimento per l’Età Evolutiva* (PROMEA; Vicari, 2007) is a measure of phonological short-term memory that requires a manipulation of phonemes without any semantic support (differently from to those tasks directly evaluating semantic access, as word repetition tests) and a phonetic-phonological planning. The task was administered following the instructions provided by the manual for this test. Participants were asked to repeat a list of 40 non-words read aloud by the examiner. Each correct answer received 1 point, for a maximum raw score of 40.

### Assessment of Working Memory

To assess working memory, the forward and backward digit span subtests of the Wechsler Scales III for children (Wechsler, 1993; Italian standardization: Orsini & Picone, 2006) were used. Following the instructions provided by the manual for this test, in the forward digit span task, the examiner asked the child to repeat in the same order the sequences of digits (s)he had just uttered while in the backward digit span task, the experimenter asked the child to repeat each sequence in the reverse order.

This task resulted in three raw scores: a *forward digit span score*, corresponding to the number of lists correctly repeated by the child in the same order pronounced by the examiner; a *backward digit span score*, representing the number of lists correctly repeated by the child in the reverse order; a *digit span total score* resulting by summing up the scores derived from the two span tasks.

### Assessment of Attention Skills

The *Modified Little Bells’* test (Biancardi & Stoppa, 1997) was used to examine participants’ selective and sustained attention. The task was administered following the instructions provided by the manual for this test. Four sheets, each including drawings of several little bells scattered among additional items, were presented to children, asking them to mark the little bells within 2 min for each sheet. They were not informed as to the time available nor the number of sheets to complete. A rapidity raw score equivalent to the total number of bells identified per sheet in the first 30 s was used to characterize the children’s selective attention. An accuracy raw score corresponding to the total number of bells found on all four sheets after the 2 min was used to measure the children’s sustained attention.

### Assessment of Theory of Mind

To assess the participants’ ability to infer other persons’ perspectives and emotions, the *Theory of Mind (ToM) Part-B* subtest from the NEPSY-II (Korkman et al., 2007; Italian standardization: Urgesi et al., 2011) was employed. ToM is multidimensional construct that is composed by a cognitive component (to infer others’ beliefs, intentions, and desires) and an affective component (to think about others’ emotional states and feelings) (Shamay-Tsoory & Aharon-Peretz, 2007). The *Theory of Mind (ToM) Part-B* subtest from the NEPSY-II assesses the affective component of theory of mind.

The task was administered following the instructions provided by the manual for this test. The children first looked at nine drawings illustrating a girl depicted from behind in several contexts (e.g., arguing with a friend, on a roller coaster) and were then asked to select among four pictures of emotional facial expressions which one might best match the girl’s expression in that specific situation. For each correct answer, children received 1 point. The first item was used as a trial. Therefore, children received a maximum raw score of 8 points.

### Statistical Analyses

To compare the performance of the two groups (ASD *vs.* TD), a series of independent *t*-tests on the variables related to non-word repetition task, attention skills, and cognitive assessment (i.e., scores obtained on tasks assessing digit forward and backward repetition, attention, and theory of mind) and on the micro- and macrolinguistic measures (i.e., number of words, number of utterances, percentage of subordinate clauses, number of settings, objects, characters, visible events, first–second-third-order connectives, temporal markers, percentage of local coherence errors, inferred events, ISTs) were performed. Moreover, for three variables (% of phonological errors, % of semantic paraphasias and % of global coherence errors) non-parametric Mann–Whitney statistics were performed. Bonferroni's correction for multiple comparisons was applied on the categories that included multiple variables: for the micro-linguistic variables (number of words, number of utterances, percentage of subordinate clauses, percentage of phonological errors, and percentage of semantic paraphasias) p < 0.001 was accepted; for the variables relating to core story details (settings, objects, characters, and visible events) p < 0.017 was accepted; for the narrative cohesion/coherence variables (first-order connectives, second-order connectives, third-order connectives, temporal markers, percentage of local coherence errors, percentage of global coherence errors) p < 0.008 was accepted. For inferred events and ISTs p < 0.05 was accepted.

To evaluate the potential relation between cognitive (forward digit span, backward digit span, and theory of mind) and micro- and macrolinguistic variables, a series of Pearson’s product-moment correlation analyses were performed within each group. Moreover, in the group of participants with a diagnosis of ASD the relations between severity scores and micro- and macrolinguistic variables were also analysed.

## Results

On non-word repetition task, attention skills, and cognitive variables (forward digit span, backward digit span, and theory of mind) the independent *t*-tests showed no significant group-related differences (Table 2). As for non-word repetition, autistic children reported a mean raw score of 34.76 (± 3.95); typically developing children reported a mean non-word repetition raw score of 33.95 (± 4.07). Raw scores of both groups were within the normative ranges of the Italian norm-reference comparison (Vicari, 2007). As for attention skills, autistic children reported a mean rapidity score of 48.73 (± 12.48) and a mean accuracy score of 114.93 (± 22.92); children of the control group reported a mean rapidity score of 48.49 (± 10.11) and a mean accuracy score of 107.73 (± 24.26). The normative ranges of the Italian standardization (Biancardi & Stoppa, 1997) for the age range 7.00–11.11 are 55.23 for the mean rapidity score and 124.15 for the accuracy score. Therefore, scores of both groups were below the normative ranges scores of the Italian standardization for this task. As for working memory, autistic children reported a mean digit span total score of 12.39 (± 2.77); typically developing children reported a mean digit span total score of 12.85 (± 3.08) (Table 2). Scores of both groups were within the normative ranges of the Italian standardization of the Wechsler Scales III for children (Orsini & Picone, 2006). As for ToM, autistic children reported a mean score of 6.41 (± 1.20); children of the control group reported a mean score of 6.17 (± 0.92). Scores of both groups were within the normative ranges of the Italian standardization of the NEPSY- II (Urgesi et al., 2011).

**Table 2:** Independent t-tests (ASD *vs.* TD group) on cognitive variables: non-word repetition score, working memory scores (forward, backward, and total digit span), selective and sustained attention score, and theory of mind. For each task, the scores are raw

	ASD M (SD) [ranges]	TD M (SD) [ranges]	*t*-test
Non-word repetition	34.76 (3.95) [22–40]	33.95 (4.07) [25–40]	*t*_(80)_ = 0.91; p = .367
Forward digit span	7.29 (1.25) [4–10]	7.88 (2.05) [5–13]	*t*_*(*80)_ = −1.56 p = .123
Backward digit span	5.10 (1.77) [0–8]	4.93 (1.66) [2–9]	*t*_(80)_ = 0.45 p = .654
Digit span total score	12.39 (2.77) [6–17]	12.85 (3.08) [9–19]	*t*_(80)_ = −.72 p = .476
Selective attention (rapidity) score	48.73 (12.48) [25–78]	48.49 (10.11) [26–75]	*t*_*(*80)_ = 0.10 p = .923
Sustained attention (accuracy) score	114.93 (22.92) [11–138]	107.73 (24.26) [45–139]	*t*_(80)_ = 1.38 p = .171
Theory of Mind	6.41 (1.20) [3–8]	6.17 (.92) [4–8]	*t*_(80)_ = 1.03 p = .306

On microlinguistic variables, a preliminary inspection of the distribution of the scores obtained by the two groups of participants on percentages of phonological errors and semantic paraphasias showed that Levene’s test was significant for these two measures (all p < 0.001). For this reason, for these two variables non-parametric Mann–Whitney statistics were performed. Overall, the two groups differed on the % of phonological errors (U = 289.50; p < 0.001) and % of semantic paraphasias (U = 493.50; p < 0.001), with the autistic group reporting more errors than the TD group. No additional significant differences emerged on the other microlinguistic variables.

On macrolinguistic variables, a preliminary inspection of the distribution of the scores obtained by the two groups of participants on percentages of global coherence errors showed that Levene’s test was significant for this measure (p < 0.001). For this reason, for this variable non-parametric Mann–Whitney statistics were performed. Overall, the two groups differed on the % of global coherence errors (U = 446.50; p < 0.001), with the autistic group reporting more errors than the TD group. For the other macrolinguistic measures, parametric statistics were performed. The independent *t*-tests showed significant differences on the number of visible events (*t*_(80)_ = −3.33; p = 0.001) and inferred events (*t*_(80)_ = −2.02; p = 0.047), with the autistic group reporting lower scores than the TD group (Table 3).

**Table 3:** Independent *t*-tests (ASD *vs.* TD group) on the narrative variables: number of settings, objects, characters, visible events, inferred events, first, second and third-order connectives, internal states terms (ISTs), temporal markers, and mistake events

			ASD M [SD]	TD M [SD]	*t*-test Mann–Whitney U
Micro-linguistic variables		Number of words	62.32 [27.41]	76.02 [36.61]	*t*_*(*80)_ = −1.92; p = .059
		Number of utterances	8.27 [2.45]	9.55 [3.13]	*t*_(80)_ = −2.04; p = .044
		% subordinate clauses	16.55 [15.53]	25.82 [19.95]	*t*_(80)_ = −2.35; p = .021
		% Phonological errors*	8.29 [7.86]	2.57 [3.59]	**U = 289.50; p < .001**
		% Semantic paraphasia*	7.61 [10.97]	1.65 [3.00]	**U = 493.50; p < .001**
Macro-linguistic variables	Core story details	Settings	1.71 [0.90]	2.12 [0.90]	*t*_(80)_ = −2.09; p = .040
		Objects	2.22 [1.21]	2.73 [1.00]	*t*_*(*80)_ = −2.08; p = .040
		Characters	4.93 [1.54]	5.37 [1.96]	*t*_(80)_ = −1.13; p = .263
		Visible events**	5.05 [1.91]	6.71 [2.55]	***t***_**(80)**_** = **−**3.33; p = .001**
	Narrative cohesion /coherence variables	First-order connectives	0.22 [0.42]	0.24 [0.54]	*t*_(80)_ = −0.23; p = .819
		Second-order connectives	0.22 [0.57]	0.17 [0.38]	*t*_(80)_ = 0.46; p = .650
		Third-order connectives	0.34 [0.57]	0.67 [1.04]	*t*_(80)_ = −1.71; p = .091
		Temporal markers	2.07 [1.68]	2.24 [1.76]	*t*_(80)_ = −0.45; p = .654
		% Errors of local coherence	39.47 [23.28]	26.78 [19.28]	*t*_(80)_ = 2.69; p = .009
		% Errors of global coherence***	12.54 [11.11]	4.49 [7.18]	**U = 446.50; p < .001**
	Inferred events		2.73 [1.50]	3.49 [1.87]	***t***_***(*80)**_** = **−**2.02; p = .047**
	ISTs		0.83 [0.95]	1.00 [1.20]	*t*_(80)_ = −0.71; p = .477

Significant differences between the two groups are given in bold

Asterisks show when the group-related difference was significant after Bonferroni correction for multiple comparisons *(p < . 001); **(p < .017); ***(p < .008)

As shown in Table 4, in the group of autistic children correlation analyses showed that the number of words was positively associated with forward (*r* = 0.41; p = 0.007) and backward (*r* = 0.39; p = 0.012) digit span; the number of utterances was positively associated with the backward digit span (*r* = 0.32; p = 0.044) and the ToM score (*r* = 0.37; p = 0.017); the percentage of phonological errors was negatively associated with backward digit span (*r* = −0.55; p < 0.001); forward digit span was positively associated with the number of settings (*r* = 0.39; p = 0.012), objects (*r* = 0.35; p = 0.024) and second-order connectives (*r* = 0.33; p = 0.036); backward digit span was positively associated with the number of settings (*r* = 0.35; p = 0.026) and objects (*r* = 0.38; p = 0.013) and negatively associated with the percentage of errors of local coherence (*r* = −0.38; p = 0.016).

**Table 4:** Correlation analyses (Pearson’s r) between cognitive and narrative variables in each group (ASD and TD)

			ASD				TD		
			ADOS severity index	Forward digit span	Backward digit span	Theory of mind	Forward digit span	Backward digit span	Theory of mind
Micro-linguistic variables		Number of words	*r* = .06 p = .711	*r* = .41 **p = .007**	*r* = .39 **p = .012**	*r* = .21 p = .187	*r* = .23 p = .150	*r* = .23 p = .148	*r* = .19 p = .238
		Number of utterances	*r* = .06 p = .690	*r* = .27 p = .091	*r* = .20 p = .207	*r* = .13 p = .415	*r* = .24 p = .131	*r* = .21 p = .189	*r* = .18 p = .272
		Percentage of subordinate clauses	*r* = −.14 p = .379	*r* = .13 p = .423	***r* = .32** **p = .044**	***r* = .37** **p = .017**	*r* = .18 p = .272	*r* = .20 p = .203	*r* = .18 p = .273
		Phonological errors	*r* = −.05 p = .747	*r* = −.27 p = .091	*r* = −.55 **p < .001**	*r* = −.30 p = .057	*r* = −.11 p = .486	*r* = −.11 p = .509	*r* = −.09 p = .569
		Semantic paraphasia	*r* = −.00 p = .998	*r* = −.23 p = .142	*r* = −.15 p = .338	*r* = .04 p = .792	*r* = −.26 p = .105	*r* = −.36 **p = .023**	*r* = .00 p = .982
Macro-linguistic variables	Core story details	Settings	*r* = −.14 p = .388	***r* = .39 ** **p = .012**	***r* = .35 ** **p = .026**	*r* = .25 p = .111	*r* = .16 p = .326	*r* = .04 p = .806	*r* = .13 p = .435
		Objects	*r* = .01 p = .961	***r* = .35 ** **p = .024**	***r* = .38 ** **p = .013**	*r* = .21 p = .188	*r* = .20 p = .232	*r* = .27 p = .084	*r* = .11 p = .512
		Characters	*r* = −.01 p = .971	*r* = .13 p = .424	*r* = .12 p = .448	*r* = .14 p = .389	*r* = −.04 p = .812	*r* = .00 p = .996	*r* = .08 p = .669
		Visible events	*r* = −.11 p = .501	*r* = .25 p = .122	*r* = .19 p = .233	*r* = .11 p = .491	*r* = .21 p = .192	*r* = .30 p = .056	*r* = .27 p = .092
	Narrative cohesion/coherence variables	First-order connectives	*r* = −.27 p = .084	*r* = .07 p = .686	*r* = .21 p = .196	*r* = .26 p = .099	***r* = .34 ** **p = .027**	***r* = .41 ** **p = .007**	*r* = .12 p = .470
		Second-order connectives	*r* = .02 p = .918	***r* = .33 ** **p = .036**	*r* = .28 p = .082	*r* = .05 p = .774	*r* = .06 p = .713	*r* = .10 p = .538	*r* = .13 p = .422
		Third-order connectives	*r* = −.08 p = .618	*r* = .10 p = .530	*r* = .14 p = .389	*r* = −.03 p = .857	*r* = .14 p = .369	***r* = .33 ** **p = .034**	*r* = .11 p = .475
		Temporal markers	*r* = .04 p = .818	*r* = .07 p = .650	*r* = .02 p = .905	*r* = −.03 p = .863	*r* = −.09 p = .582	*r* = .01 p = .927	*r* = −.04 p = .795
		Errors of local coherence	*r* = .10 p = .546	*r* = −14 p = .376	*r* = −.38 **p = .016**	*r* = −.08 p = .609	*r* = −.23 p = .140	*r* = −.35 **p = .026**	*r* = −.14 p = .384
		Errors of global coherence	*r* = .20 p = .203	*r* = .15 p = .360	*r* = −.07 p = .678	*r* = −.11 p = .479	*r* = .12 p = .470	*r* = .23 p = .154	*r* = .00 p = .999
	Inferred events		*r* = −.07 p = .667	*r* = .07 p = .666	*r* = .18 p = .262	*r* = .09 p = .572	*r* = .20 p = .199	*r* = .27 p = .090	*r* = .26 p = .107
	ISTs		*r* = −.15 p = .339	*r* = −.02 p = .901	*r* = .16 p = .320	*r* = .11 p = .503	*r* = .21 p = .182	*r* = .10 p = .535	*r* = .14 p = .398

Significant differences between the two groups are given in bold

In the group of children with TD, correlation analyses showed that the percentage of semantic paraphasias was negatively associated with backward digit span (*r* = -0.36; p = 0.023); forward digit span was positively associated with the number of first-order connectives (*r* = 0.34; p = 0.027); backward digit span was positively associated with the number of first-order connectives (*r* = 0.41; p = 0.007) and third-order connectives (*r* = 0.33; p = 0.034) and negatively associated with the percentage of errors of local coherence (*r* = -0.35; p = 0.026). (Table 4).

### Summary of the Results

Overall, the results suggest that autistic children included in their stories a fewer number of visible events and inferred events than children of the control group. Moreover, the stories generated by children in the autism spectrum contained more phonological errors and semantic paraphasias (lexical errors) than the stories produced by children with typical development as well as more errors of global coherence. As for correlation analyses, in both groups significant correlations were found between some narrative variables (both micro- and macrolinguistic) and components of working memory, which therefore resulted as the cognitive process mostly associated with story generation. ToM was, in fact, positively associated with one variable, i.e., the percentage of subordinate clauses, only in the autistic group.

## Discussion

In the present exploratory study, we examined storytelling skills in a cohort of autistic children and children with typical development by applying a multilevel procedure of discourse analysis which includes both micro- and macrolinguistic measures. From a microlinguistic point of view, the results revealed that autistic children produced stories with higher percentages of phonological and lexical errors than the narratives produced by children of the control group. For what concerns the macrolinguistic measures, autistic children included in their narratives a fewer number of visible events and inferred events than children of the control group. Moreover, the stories of the participants on the autism spectrum were less coherently organized than the narratives of the participants of the control group because they contained a higher percentage of errors of global coherence. These results corroborate previous investigations attesting significant narrative production difficulties in autistic children (e.g., Ferretti et al., 2018; Greco et al., 2023; Kenan et al., 2019; King et al., 2013, 2014; Marini et al., 2019, 2020; Peristeri et al., 2017; Rumpf et al., 2012) and have important theoretical and clinical implications.

### Microlinguistic Variables

From a microlinguistic (i.e., phonological, lexical, and grammatical) point of view, our results highlighted that, when producing a story, children on the autism spectrum generate narratives that were comparable in terms of narrative length (number of words and number of utterances) and syntactic complexity to those produced by the children of the control group but with more phonological and lexical errors. On these variables the literature reports conflicting findings. Some investigations pointed out that autistic children tend indeed to produce impoverished narratives in terms of both verbal productivity (e.g., story length) and complexity of syntax (Greco et al., 2023; Kenan et al., 2019; King et al., 2013; Marini et al., 2020; Peristeri et al., 2017; Rumpf et al., 2012; Tager-Flusberg, 1995). However, other studies (e.g., Diehl et al., 2006; Tager-Flusberg & Sullivan, 1995) indicated that, when autistic groups and control groups are matched on language ability, many of these differences vanish. In this respect, it should be highlighted that in our study the two groups were administered a non-word repetition task to control for language ability. Indeed, according to several studies (Harper-Hill et al., 2013; Kjelgaard & Tager-Flusberg, 2001; Marini et al., 2020) and consensus conferences (e.g., Sansavini et al., 2021), this task is highly sensitive to linguistic impairments in children. Research by Marini et al. (2020) showed that autistic children who scored lower than children with typical development on a non-word repetition task also obtained the lowest scores on morphological, lexical, grammatical, and narrative measures. The autistic children included in the present investigation performed similarly to the children of the control group on the non-word repetition task. Despite that, group differences on phonological and lexical errors were found, with the participants of the autistic group producing more errors than the children of the control group. This result might suggest that the two groups were not matched for structural language abilities. This might have important clinical implications. In fact, differently from previous studies (Harper-Hill et al., 2013; Kjelgaard & Tager-Flusberg, 2001; Marini et al., 2020), our finding does not appear to support the efficacy of non-word repetition tasks in detecting the presence of linguistic differences between autistic children and children of the control group. Future investigations should take this into consideration when using non-word repetition task to control for language competence in children on the autism spectrum. That said, a limitation of the present study is that no other tasks traditionally employed for a comprehensive assessment of linguistic abilities (e.g., sentences completion or lexical naming) have been administered to the two groups.

Correlation analyses showed that in the autistic group the two components of working memory were associated with several microlinguistic measures, among which the number of words (positively correlated with backward and forward digit span) and syntactic complexity (positively correlated with backward digit span). These correlations are in line with previous findings (Adornetti et al., submitted; Kuijper et al., 2017; Tsimpli et al., 2014) and may suggest that both the production of new words and subordinate clauses, which requires a short-term connection between ideas, could reflect the maintenance and manipulation of short-term information as that involved in backward and forward digit span.

Particularly interesting appears the positive correlation between the percentage of subordinate clauses and ToM scores in autistic children. This result is consistent with findings by Kuijper et al. (2017) who also showed that ToM is associated with measures of syntactic complexity during narrative production. More in general, such result agrees with previous research suggesting a relation between linguistic abilities, e.g., those necessary to generate subordinate clauses introduced by the complementizer “that’’, and mentalizing abilities (e.g., de Villiers & Pyers, 2002; Ebert, 2015; Hale & Tager-Flusberg, 2003). A longitudinal study in autistic children by Tager-Flusberg and Joseph (2005) found that mastering sentential complements significantly predicted performance on ToM task one year later in 5 to 14-year-old participants. From a clinical point of view, our finding therefore provides support to intervention protocols aimed to train sentential complements of communication verbs, such as tell, say, speak (e.g., X says that), to improve performance in ToM of both autistic and typically developing children (e.g., Durrleman et al., 2022).

### Macrolinguistic Variables

Results emerging from the macrolinguistic assessment provide further evidence of the difficulties affecting autistic children when they are faced with the conceptual organization of the macrostructure of a story. As for the analysis of core story details, results showed that, compared to participants of the control group, autistic children included in their stories fewer units of information involving visible events. This finding is in line with results from previous investigations that analysed story’s basic components (Banney et al., 2015; Kenan et al., 2019; Peristeri et al., 2017; Rumpf et al., 2012; Suh et al., 2014). A study by Suh et al. (2014) compared the narratives produced by children and adolescents on the autism spectrum with those generated by a control group of typically developing peers. The participants were asked to listen to the examiner telling the beginning of the narrative depicted in the *Tuesday* book (Wiesner, 1991) and then were encouraged to complete the story. The narratives produced by the participants were analysed in reference to several variables, among which the number of story elements, namely “the events representing the ‘essential features’ of the narratives” (p. 1686). As in the present investigation, analyses revealed that autistic participants produced significantly fewer story elements than participants with typical development. Similar results were found by Kenan et al. (2019), who examined storytelling abilities in 24 children with a diagnosis of ASD (all boys) using the *Tuesday* narrative (Wiesner, 1991). Differently from Suh et al. (2014), in the study by Kenan et al. (2019) participants were asked to tell the entire story when looking at the pictures. Results confirmed that the stories generated by autistic children contained fewer actions (that were comparable to the visible events of the current research) than those produced by the control group of TD peers. Kenan et al. (2019) also found group differences in the number of settings and characters, with the autistic children introducing in their narratives a significantly fewer number of these elements than participants of the control group. We did not find group differences on these variables. The younger age range of the participants of that study (4.10–7.0) as compared with the participants in the current investigation (7.02–11.03) may explain these different results.

The analysis focusing on measures of cohesion and coherence showed that the stories produced by the two groups were similar in terms of connectives marking the temporal and causal relations between sentences as well as in reference to the percentage of errors of local coherence, but they differed as for the percentage of errors of global coherence. Regarding the use of temporal and causal connectives, the literature reports inconsistent findings. For instance, Tager-Flusberg (1995) showed that autistic children had difficulty using causal statements to explain the causal relationship between events in the stories. Ferretti et al. (2018) found that children on the autism spectrum included in their fictional narratives a fewer number of causal links than peers with typical development. In contrast, Tager-Flusberg and Sullivan (1995), Capps et al. (2000), Suh et al. (2014) did not find differences between autistic participants and individuals of the control groups as for the use of causal references in narratives. As highlighted by Sah and Torng (2015), these conflicting findings might be attributed to methodological issues, e.g., to the different tasks employed to elicit narrative production as well as to the selection of groups. For example, in the study by Ferretti et al. (2018) the narrative task required participants to tell an imagined story, only providing them with prompts, i.e., the pictures of the first or the last event of that story. On the contrary, in the present study the children could see the pictures of the story’s events when generating their narratives. Therefore, while in Ferretti et al. (2018) investigation children had to imagine new sequences of events as well as new causal links connecting them, in the current study children could see how the events constituting the narrative were linked. Therefore, it is possible that tasks employing sequences of pictured cards to elicit narrative makes the process of building causal connections between events simpler than other narrative tasks, such as those based on prompts.

As for the selection of groups, autistic participants of the study by Tager-Flusberg (1995) had lower cognitive abilities, which makes it difficult to determine whether the differences observed could be the result of impairment in intellectual functioning or were specific for ASD. In the study by Ferretti et al. (2018) autistic children had lower scores on tasks assessing episodic future thinking (i.e., the ability to mentally project themselves into the future) and working memory than children with typical development. In the present investigation, the two groups were matched for cognitive abilities such as working memory and ToM as well as for phonological short-term and attention skills. This might contribute to explain the similar performance of the two groups on these measures as well as the similar scores on errors of local coherence. Supporting this interpretation, in fact, correlations analyses showed that backward digit span was negatively associated with the percentage of errors of local coherence in both groups (the more the children obtained higher scores on backward digit span the fewer errors of local coherence they made). From this view, working memory abilities within the normative ranges seem associated to the building of coherently connected adjacent utterances: it can be hypothesized that linking adjacent utterances, needing a short-term connection between ideas, reflects keeping track of the various aspects of the events that have to be connected. This result is in line with previous research showing that working memory has an important role in children's narrative development (e.g., Dodwell & Bavin, 2008; Veraksa et al., 2020; Ward et al., 2016).

Differences between the two groups emerged on global coherence, with the autistic group producing more errors than the control group. This finding agrees with results of some of the previous studies, suggesting that autistic individuals tend to generate less coherent spoken narratives than the control groups (e.g., Baixauli et al., 2016; Ferretti et al., 2018; Harvey et al., 2023; Marini et al., 2020; Sah & Torng, 2015). It should be highlighted that in the literature different types of narrative coherence scoring schemes were employed. As pointed out in the systematic scoping review by Harvey et al. (2023, Fig. 2, p. 7), at least seven different approaches to analyzing narrative coherence in autistic persons can be identified. Some studies investigated global coherence in terms of causal connectedness of a narrative (Diehl et al., 2006; Sah & Torng, 2015), with higher scores indicating a greater level of connectedness. Others, such as Brown (2007), employed a version of the Narrative Coherence Coding Scheme (Reese et al., 2011), which splits global coherence into three dimensions: context, chronology, and theme. Depending on the level of elaboration, each dimension is rated on a 0–3 scale. In the current study, we assessed narrative coherence in terms of incongruence (Harvey et al., 2023), i.e., errors of global coherence − tangential, incongruent, repetitive and fillers utterances. Our results agree with findings from previous investigations that employed the same scoring scheme (Marini et al., 2020) or that assessed the presence of irrelevant details and illogical, redundant, or inappropriate utterances in the narratives of autistic people (Ferretti et al., 2018; Kauschke et al., 2016; Losh & Capps, 2003; Mäkinen et al., 2014). Overall, such result provides further support to the view that managing the global representation of story content is particularly challenging for autistic people. However, it should be noted that the sequence of picture cards used to elicit narrative production is intended to lead to a linear story creation − the construction of a global representation of story content is constrained by linearity. This is not the narrative style found in all cultures (e.g., Carmiol & Sparks, 2014). This is a point that should be considered when comparing results of studies from different cultural backgrounds.

Expanding previous narrative assessments (e.g., Kenan et al., 2019; Marini et al., 2020; Peristeri et al., 2017), we also included in our analysis a measure relating to inferred events. The ability to infer information not explicitly stated in a text (or depicted in a visual narrative) is indeed a crucial factor for the construction of the mental model of a story (Graesser et al., 1994). Several studies reported impoverished inferential ability in ASD (e.g., Cardillo et al., 2021; Dennis et al., 2001; Norbury & Bishop, 2002). In line with these studies, from our results emerged that autistic children included in their stories fewer references to inferred events than participants with typical development, thus pointing to a difficulty in representing pieces of information not depicted in the visual stimuli, but necessary to give meaning to visible events.

Lastly, it should be highlighted that we did not find group differences on internal states terms. This finding is consistent with many of the previous studies (Bang et al., 2013; Capps et al., 2000; Tager-Flusberg & Sullivan, 1995) but contrast with Tager-Flusberg (1995) and, more recently, with the investigation by Kenan et al. (2019). These discrepancies might be explained by the different age range of the children of these studies. The autistic children autism in Tager-Flusberg’s study aged from 3,4 to 7,7 (and had delayed language development); Kenan et al. (2019) administered the task to children aged from 4 to 7 years. Developmental research on ToM indicates that important changes in this cognitive ability take place at around age 4 in children with TD (e.g., Wellman & Lagattuta, 2000; Wellman et al., 2001; Wimmer & Perner, 1983) while in autistic children there is both a delayed onset and a slower development at varying rates in ToM (Broekhof et al., 2015; Pino et al., 2017). Since our study included children aged 7–11 years, it is likely that the mindreading ability required to narrate the “Nest story” was within the reach of autistic children of this age range and, indeed, the two groups obtained similar scores. In addition, these discrepancies might also be related to methodological issues, such as the different stimuli used to elicit the story. In fact, the “Nest story” includes only one event that is susceptible to mentalistic interpretation, and it probably does not work as an effective tool for assessing the use of mental state terms in clinical populations. This is a limitation of the present study that future research should consider.

Overall, the results of the current exploratory study might have important clinical implications in that they point to the relevance of developing training programs aimed to improve specific aspects of narrative production. Specifically, our study bolsters previous research aimed to improve story structure planning, i.e., character, setting, internal response, etc. (Favot et al., 2018), as well as the use of complex syntax along with temporal and causal connections that can help autistic children to construct coherent stories (Gillam et al., 2015; Hilviu et al., 2023; Petersen et al., 2014).

### Limitations

Some limitations of the current exploratory study need to be highlighted. As already mentioned, although we employed a non-word repetition task to control for possible linguistic differences between autistic children and children of the control group, this task turned out not to provide a reliable assessment of language competence. Thus, inclusion of a comprehensive language test would have allowed us to better determine the linguistic profile of the two groups. Moreover, it should be acknowledged that the ToM task assessed only one component of such ability—the affective one. Inclusion of a task also measuring the cognitive component—e.g., a false belief task—would have provided a more accurate evaluation of mindreading abilities. Related to this, the narrative text turned out not to be particularly adequate to evaluate the use of mental states terms in story production.

That said, the current research has several strengths. The sample group was larger than previous studies. As pointed out in the systematic scoping review by Harvey et al. (2023), only 12% of studies (out of 59) on narrative in ASD had a sample of more than 40 autistic participants. For this reason, also considering that the two groups were strictly matched regarding cognitive measures such as working memory and affective theory of mind and that the narrative coding system combined multiple aspects of story production, this investigation contributes to provide solid data on the storytelling abilities of autistic children.

## Conclusion

The present study shows that even autistic children with adequate cognitive skills display several differences in their narrative competence affecting both micro- and macrolinguistic aspects of story production. The narratives produced by autistic children contained more phonological and lexical errors as well as more errors of global coherence than the stories generated by participants of the control group. Moreover, children on the autism spectrum included in their narratives a fewer number of events (both visible and inferred) than their typically developing peers. From a clinical point of view, this study proves the efficacy of using procedures of narrative discourse assessment to appropriately describe the linguistic and narrative profile of autistic children that can possibly lead the development of narrative treatments aimed to improve specific aspects of story production.

## Data Availability

The datasets generated during and/or analyzed during the current study are available from the corresponding author on reasonable request.
